# A Review of Topical Diclofenac Use in Musculoskeletal Disease

**DOI:** 10.3390/ph3061892

**Published:** 2010-06-11

**Authors:** Bindu Nair, Regina Taylor-Gjevre

**Affiliations:** Division of Rheumatology, University of Saskatchewan, Royal University Hospital, Saskatoon, Saskatchewan S7N 0W8, Canada; E-Mail: Bindu.Nair@saskatoonhealthregion.ca (B.N.)

**Keywords:** NSAIDs, musculoskeletal, osteoarthritis, diclofenac

## Abstract

Nonsteroidal anti-inflammatory drugs (NSAIDs) are commonly prescribed medications for the treatment of musculoskeletal disorders. Osteoarthritis is the most common form of arthritis in humans and its prevalence rises with age. Oral NSAIDs have potential associated toxicities that must be monitored for and can limit the use of these drugs in certain populations including people of older age. Topical NSAIDs are now being recognized as an option for the treatment strategy of osteoarthritis. We review the efficacy and safety of one of the most common topical NSAIDS, topical diclofenac, for the treatment of osteoarthritis.

## 1. Introduction

Non-steroidal anti-inflammatory drugs (NSAIDs) are widely employed in musculoskeletal disease, both for their anti-inflammatory as well as their analgesic properties. Use of these agents may extend from the acute injury to the long-term chronic disorder, and includes conditions considered to be 'degenerative' as well as those of a clearly inflammatory etiology. Efficacy of various NSAIDs in different clinical settings has been extensively evaluated [1,2,3]. In rheumatologic practice, they are commonly employed as monotherapy in treatment of osteoarthritis.

Osteoarthritis (OA), also known as osteoarthrosis or degenerative joint disease, is the most common form of arthritis. OA is a common cause of long-term disability in the adult population [4]. Prevalence varies among populations, but increases universally with age. Knee OA is evident radiographically in an estimated one fifth of people beyond the age of 65 years. A proportion of these will be symptomatic and will progress to disability [5,6]. Hand OA typically affects the first carpometacarpal joint as well as the distal and proximal interphalangeal joints. X-ray changes of osteoarthritis have been seen in 22.1% of hand joints in men and 32.7% in women over the age of 70 [7]. With an aging population OA will continue to have a major socioeconomic impact in North America. 

Osteoarthritis may be considered a group of overlapping distinct diseases which may have different etiologies but with similar biologic, morphologic, and clinical outcomes [8]. There are many epidemiologic associations with increased risk for OA including trauma, continuous overuse, obesity, gender as well as certain metabolic, collagen or endocrine disorders. Early in the disease process the articular cartilage will become edematous and develop irregularities and microscopic cracks. The chondrocyte response results in increased type 1 and 3 collagen production. The collagen fibrils become loosely packed and fragmented. There is increased intra-articular release of degradative enzymes including matrix metalloproteinases, collagenase, gelatinase and stromelysin. Over time, these enzymes produce a dominantly catabolic state. Diminished proteoglycan content of the cartilage is followed by thinning and increased fissuring in the cartilage layer. Eventually denudation of the subchondral bone develops. Synovial fluid may be forced by mechanical influences into the subchondral bone forming cystic structures. Remodeling and repair mechanisms throughout this process result in new bone formation, subchondral sclerosis and osteophytes [9]. Abnormal signal consistent with edema in the adjacent bone marrow has been visualized by MRI in symptomatic OA patients [10].

In symptomatic OA patients pain is the most common symptom, contributing to functional limitations and disability. The source of the pain is uncertain, as cartilage itself is uninnervated. Creamer *et al.* found 40% of symptomatic OA patients did not achieve relief with intrarticular anesthetic [11], suggesting an extra-articular source of pain in some patients. Two potential extra-articular sites would include the soft tissues, the bursae, muscles, tendons and ligaments, adjacent to the afflicted joint and secondly, the bone marrow which is rich in sensory fibers. Evidence for involvement of both of these sites in pain production in OA has been recognized [12,13,14]. NSAIDs are recommended for the management of Osteoarthritis by the Osteoarthritis Research Society International (OARSI), the American College of Rheumatology (ACR), and the European League Against Rheumatism (EULAR) [15,16,17].

Unfortunately, there are clinical circumstances in which caution is required in use of NSAIDs. Particularly in the elderly patient, the patient with multiple co-morbidities, or the patient with chronic musculoskeletal disease where the expectation is one of prolonged use of NSAIDs. Concern for renal, hepatic and gastrointestinal toxicity is highly appropriate in such situations. Oral NSAIDs are used extremely cautiously if at all in patients with renal insufficiency, congestive heart failure, hypertension, and various forms of liver disease [18,19,20] 

## 2. Topical NSAIDs

Topical NSAID preparations were developed for local application. The rationale for development of this targeted delivery method was essentially to decrease systemic absorption and potentially thereby limit toxicity without sacrificing local effect and benefit.

The dermis of the skin is rich in high molecular weight proteoglycans which are hydrophobic and allow for uptake of water soluble medications. Additionally, the dense capillary and lymphatic network allows for some penetration to deeper subcutaneous fatty tissue where lipophilic agents may accumulate. Systemic penetration of topical agents is dependent on liposolubility, molecular weight, partial charge of the molecule, aqueous solubility, the presence of certain functional groups on the drug molecule and kinetics of blood flow with reference to relative anatomic vascularity [21]. For optimal efficacy, the NSAID has to penetrate to the inflamed tissue in a concentration adequate to exert meaningful anti-inflammatory activity. The mechanism of anti-inflammatory action in based on the COX enzyme inhibition by NSAID class agents.

Several NSAID formulations have been available in topical form including: diclofenac preparations, ketoprofen gel, piroxicam patch/cream and ibuprofen cream/gel among others [22]. Efficacy comparisons between topical formulations have been minimally evaluated [23]. Diclofenac has however been the most widely studied in reference to musculoskeletal disorders. Topical diclofenac is felt to reduce inflammation by inhibition of the COX isoenzymes and thereby decreasing synthesis of proinflammatory prostaglandins. The analgesic effect of topical diclofenac is not fully understood. At high tissue concentrations diclofenac appears to have the capacity to act as a sodium channel blocker to mediate local-anesthetic like effects on nociceptive afferent fibers [24]. Animal studies have suggested recently that peripheral NMDA receptor antagonism may contribute to analgesic effects of locally administered diclofenac [25]. There has also been some evidence that diclofenac may inhibit L-type calcium channels which participate in pain perception [26]. 

Transdermal penetration of diclofenac may be variable [27]. Various salts of diclofenac have been investigated for their topical absorptive properties. The inclusion of percutaneous enhancers, solvent compositions and rheological properties have been shown to be important. Microemulsion formulations and preparations containing penetration enhancers such as dimethyl sulfoxide (DMSO) have been studied and developed to promote topical absorption of diclofenac [21]. In animal models, iontophoresis in conjuction with geraniol has been reported to be an effective transdermal delivery system [28]. Evaluation in animal models of the effect of vehicle on topical diclofenac penetration may lead to future expansion of therapeutic choices [29,30,31]. 

Diclofenac has been available in several different topical formulations, available in different jurisdictions. These include diclofenac sodium 1% gel, diclofenac diethylamine gel 1.16%, MIKA diclofenac spray 4% gel, diclofenac DMSO lotion, and diclofenac epolamine (diclofenac hydroxyethyl-pyrrolidine) patch. Metabolism of diclofenac occurs primarily in the liver, and the majority is eliminated in the urine. In healthy volunteers, the mean terminal elimination half-life was 88.4 hours [32].

In a comparison of systemic bioavailability between topical diclofenac sodium 1% gel compared to oral diclofenac sodium in normal volunteers, Kienzler has demonstrated a 5-17 fold lower systemic absorption with the topical preparation [33]. Topical application has also been shown to result in higher concentrations in adjacent adipose tissue and skeletal muscle than oral preparations [34,35]. However, synovial concentrations with topical diclofenac were lower than with oral use [35]. It has been recently suggested that dermal oxidative stress from UV radiation may impede absorption of topical diclofenac, although, a clinical trial in patients with sunburn did not show any significant increase or decrease in absorption measures [36,37].

## 3. Efficacy in Musculoskeletal Disease

The features of ten trials studying the efficacy of topical diclofenac treatment in osteoarthritis of the knee are presented in Table 1. All of the studies are double blind, randomized trials with their treatment arm consisting of several formulations of topical diclofenac, either as a patch, solution or gel [38,39,40,41,42,43,44,45,46,47]. 

**Table 1:** Features of Studies of Topical Diclofenac Treatment for Knee Osteoarthritis.

Study	Design	Sample, N	Duration	Treatment	Dosing	Control
Dreiser 1993 [38]	Double blind, randomized	155	15 days	Diclofenac hydroxyethylpyrrolidine (DHEP) plasters	180 mg bid	Placebo plaster
Grace 1999 [40]	Double blind, randomized	70	2 weeks	2% diclofenac in lecithin organogel	2.5 g tid	Placebo
Bruhlmann 2003 [39]	Double blind, randomized	103	2 weeks	DHEP patch	180 mg bid	Placebo patch
Bookman 2004 [43]	Double blind, randomized	248	4 weeks	1.15% diclofenac/45.5% DMSO	1.3 mL qid	Placebo, vehicle (45.5% DMSO)
Roth 2004 [44]	Double blind, randomized	326	12 weeks	1.15% diclofenac/45.5% DMSO	1.3 mL qid	Vehicle
Tugwell 2004 [46]	Double blind, double dummy, randomized	622	12 weeks	1.5% diclofenac/45.5% DMSO	1.55 mL tid	Placebo oral tablets, placebo topical solution, diclofenac po 50 mg tid
Baer 2005 [47]	Double blind, randomized	216	6 weeks	1.5% diclofenac solution/45.5% DMSO	1.3 mL qid	Vehicle
Niethard 2005 [41]	Double blind, randomized	238	3 weeks	1.16% diclofenac diethylamine gel	4 g qid	Placebo gel
Barthel 2009 [42]	Double blind, randomized	492	12 weeks	1% diclofenac sodium gel	4 g qid	Vehicle
Simon 2009 [45]	Double blind, double dummy, randomized	775	12 weeks	1.15% diclofenac/45.5% DMSO	1.2 mL qid	Placebo solution, DMSO vehicle, Oral diclofenac (100 mg SR)

All of the studies used in their outcome assessments measures of pain, physical function and some type of patient derived rating of disease or efficacy. Dreiser *et al.* looked at diclofenac plaster application and found that by day 4 there were already differences in favor of the treatment arm in improvement in pain and functional impairment [38]. Bruhlman *et al.* also assessed the efficacy of an adhesive vehicle to carry the diclofenac formulation and found significant improvement in outcome assessments of pain and physical function compared to placebo at days 7 and 14. However there was no significant difference between topical diclofenac and placebo groups in terms of the walking time assessment [39]. The data from these two studies was later studied in a pooled analysis and the authors calculated that the number need to treat (NNT) with diclofenac plaster for at least a 50% reduction of pain was 3.0 [48].

A 2% diclofenac lecithin organogel formulation was studied by Grace *et al*. with the topical NSAID gel also achieving significant improvement from baseline assessment as well as in comparison to the control group by the end of the two week study [40]. Niethard *et al.* assessed 1.16% diclofenac diethylamine gel compared to placebo gel and found improvements in efficacy endpoints peaked at week 2 and maintained up to the 3rd week [41]. Barthel *et al.* assessed 1% diclofenac sodium gel against its vehicle with 492 patients in a 3 month study. All efficacy outcomes for the treatment arm were superior to the vehicle at the end of the 12 weeks including the WOMAC pain, WOMAC function and global rating of the disease. The differences between the two groups were noted in the first week of the study. The contralateral knee was assessed with the WOMAC pain subscale and did not show a substantial difference in the pain scores from baseline to week 12 in either the treatment or control groups [42].

Topical diclofenac solution efficacy for osteoarthritis of the knee has been reported in several efficacy studies ranging from 4, 6 and 12 weeks in duration [43,44,45] (Table 2). Bookman *et al.* showed significant improvement for knee osteoarthritis with 1.15% diclofenac sodium in a carrier containing DMSO (dimethyl sulfoxide) compared to the carrier containing DMSO as well as to a placebo solution containing a token amount of DMSO. The topical diclofenac arm showed an improvement of 42.9% for pain, 39.3% for physical function, 40.5% for stiffness and 44.45% for pain on walking [43]. Roth *et al.* showed that the improvements with the topical diclofenac solution continued for up to 12 weeks compared to the vehicle control arm [44]. Simon *et al.* conducted a five arm study with 775 patients for a 12 week period. Patients were randomized to either the topical diclofenac solution with a placebo solution, DMSO vehicle, oral diclofenac and combination of topical and oral diclofenac. The topical diclofenac solution was superior in the efficacy outcomes for pain, physical function and patient global assessment as compared to the placebo solution and the DMSO vehicle. There was no significant difference between the topical diclofenac and the oral diclofenac. The combination of topical and oral diclofenac did not show any advantage over oral diclofenac in the outcome assessments [45]. Tugwell compared topical diclofenac with oral diclofenac in an equivalence study of the treatment of osteoarthritis of the knee. At the end of the 12 week trial, no difference was found between the difference in mean change scores (final minus baseline) between the two arms of the study in the WOMAC pain score, physical function score as well as the patient global assessment [46].

**Table 2:** Results of studies of topical diclofenac treatment for knee osteoarthritis.

Study	Outcome Assessment	Results, compared to control arm, topical diclofenac showed:
Dreiser 1993 [38]	Spontaneous pain (Huskisson's analogical scale)	More reduction in pain (Huskisson's test -33.7 *vs.* 22.4, p < 0.002).
	*Lequesne's index	Greater decrease in Lequesne's index (-5.0 *vs.* -2.8, p < 0.001).
	Patient rated global efficacy	Better patient global efficacy.
Grace 1999 [40]	**Western Ontario and McMaster Universities (WOMAC) Osteoarthritis Index	More reduction in WOMAC score, and pain and physical function subscores (-12.63 *vs.* -3.30, -16.49 *vs.* -4.35 and -11.96 *vs.* -3.17, p = 0.05).
Bruhlmann 2003 [39]	Spontaneous pain	Significant improvement in spontaneous pain (2.1 *vs.* 3.9, p < 0.01).
	Lequesne's algofunctional index	More improvement with Lequesne's index at days 7 (8.0 *vs.* 9.5, p < 0.05) and 14 (6.9 *vs.* 9.0, p < 0.01).
	Physician's and patient's global assessment of efficacy	Physician's efficacy rated excellent in 10.2% of DHEP patch group while same rating in 8.9% of placebo cases. 24.5% patients consider DHEP patch to be excellent, same rating given to placebo in 8.9% of patients.
Bookman 2004 [43]	WOMAC pain, physical function and stiffness scores	More reduction in pain (-3.9 *vs.* -2.5 and -2.5, p = 0.023 and 0.016), physical function (-11.6 *vs.* -5.7 and -7.1, p = 0.002 and 0.014) and stiffness (-1.5 *vs.* -0.7 and -0.6, p = 0.015 and 0.002).
	Pain on walking	Improvement in pain on walking (-0.8 *vs.* -0.4 and -0.6, p = 0.003 and 0.015).
	Patient global assessment (PGA)	Better PGA score (6.7 *vs.* 7.8 and 7.8, p = 0.039 and 0.025)
Roth 2004 [44]	WOMAC	More reduction in WOMAC pain (-5.9 *vs.* -4.3, p = 0.001), physical function (-15.4 *vs.* -10.1, p = 0.002).
	PGA	Better PGA score (-1.3 *vs.* -0.9, p = 0.002).
	Pain on walking	More improvement in pain on walking (-1.18 *vs.* -0.87, p = 0.005).
Tugwell 2004 [46]	WOMAC pain and physical function	No significant difference in WOMAC scores.
	PGA by visual analogue scale (VAS)	No difference in PGA.
Baer 2005 [47]	WOMAC	Greater change in pain (mean -5.2 *vs.* -3.3, p = 0.003), physical function (-13.4 *vs.* -6.9, p = 0.001).
	PGA	More change in PGA (-1.3 to -0.7, p = 0.0001).
Niethard 2005 [41]	End of day pain on movement (VAS)	More improvement over days 1-14 (difference 4 mm, p = 0.02) and days 8-21 (difference 6 mm, p = 0.005).
	WOMAC	Difference 9 points for pain (p = 0.0002), 9 points for stiffness (p = 0.0004) and 8 points for physical function indices at week 3 (p = 0.001).
	Pain intensity (VAS)	More improvement in pain efficacy (difference 6 mm at week 1, p = 0.03; 11 mm at week 2, p = 0.0002, 9 mm at week 3, p = -0.006).
	^OARSI/OMERACT	Better response rate (62% *vs.* 46%, p = 0.01).
	Patient assessment of efficacy	Greater rated patient assessment of efficacy
Barthel 2009 [42]	WOMAC pain and physical function subscales	More improvement in pain (-5.0 *vs.* -4.0, p = 0.01) and physical function (-15.0 *vs.* -10.9, p = 0.001).
	Global rating of disease	More improvement in mean global rating (-27.0 *vs.* -18.2 p < 0.001)
Simon 2009 [45]	WOMAC pain and physical function	Improvement in change compared to placebo for pain (-6.0 *vs.* -4.7, p = 0.015), physical function (-15.8 *vs.* -12.3, p = 0.034). Also more improvement compared to DSMO. No difference compared to oral Diclofenac.
	Patient Overall Health Assessment (POHA)	Improvement in change compared to placebo (-0.95 *vs.* -0.37, p < 0.0001). Also more improvement compared to DSMO. No difference compared to oral Diclofenac.
	WOMAC stiffness	No difference compared to placebo. More improvement compared to DSMO. No difference compared to oral Diclofenac.
	PGA	Improvement in change compared to placebo (-1.36 *vs.* -1.01, p = 0.016). Also more improvement compared to DSMO. No difference compared to oral Diclofenac.

* The Lequesne index has questions pertaining to pain or discomfort, maximum distance walked, and activities of daily living. The total questionnaire is scored on a 0 to 24 scale, with lower scores meaning less functional impairment [49]. ** The WOMAC Osteoarthritis Index is a questionnaire consisting of 24 questions covering the domains of pain, physical function and stiffness with each response scored on a 5 point Likert scale (0–4 with 0 representing none) [50]. ^ The OARSI/OMERACT response rate (Osteoarthritis Research Society/Outcome Measures in Rheumatology) which includes three symptom domains of pain, physical function and patient global assessment [51].

In evaluation of treatment of primary osteoarthritis of the hand, Altman *et al.* carried out a randomized, double-blind controlled trial looking at the efficacy of 1% diclofenac sodium gel compared to placebo (vehicle) in 385 patients [52]. Diclofenac sodium gel was associated with 42–45% reduction in visual analogue scale (VAS) pain intensity, 35–40% reduction in total Australian/Canadian Osteoarthritis Hand Index (AUSCAN) score and 36–40% reduction in global rating of disease after 4 and 6 weeks. At week 8, there was still a trend to more improvement in the treatment arm but no statistically significant with VAS pain intensity and global rating of disease. At week 8, the total AUSCAN score of the diclofenac sodium gel group was significantly superior to the vehicle group. The mean level of compliance was found to be greater than 75% in every week of the study. Zacher *et al.* carried out a study for osteoarthritis of the finger joints (Heberden and/or Bouchard arthritis) in 155 patients comparing the efficacy of 1.16% diclofenac diethylamine gel 10 cm ribbon qid *versus* oral ibuprofen 400 mg tid. After 3 weeks, the double blind, randomized study demonstrated with the response rate that the diclofenac gel as at least as effective as the oral ibuprofen [53]. 

Topical diclofenac has been studied in the treatment of musculoskeletal disorders other than osteoarthritis. Mueller *et al.* investigated the extent and time course of pain intensity with the treatment of a diclofenac patch compared to placebo in acute traumatic sport injury in 120 subjects over a 1 week period. The diclofenac patch showed more relief of pain than the placebo with the greatest response on day 2 and day 3 [54] Hsieh *et al.* looked at myofascial pain syndrome in the upper trapezius treatment in 153 patients randomized to either a diclofenac patch or a control (menthol) patch for 8 days. The diclofenac patch group showed an improvement compared to the control patch group in pain response as well as cervical active range of motion [55].

## 4. Adverse Effects

Adverse effects have been reported with topical diclofenac. Zimmerman reported a case series of four patients with gastrointestinal (GI) bleeding after initiation of topical diclofenac diethylammonium 1.6% in a fatty emulsion base in aqueous gel [56]. These four cases were among 110 patients admitted for upper GI hemorrhage to a single hospital in one year. Of the four patients, two used the topical diclofenac for relief of back pain that was incorrectly attributed to musculoskeletal etiology but more likely referred pain from gastric ulcer disease. The remaining two patients had pre-existing history of GI ulcer disease before using the topical diclofenac gel. The authors of the case series suggest that use of the topical diclofenac emulsion gel be used cautiously in patients with known history of GI ulcers [56].

In most clinical trials employing topical diclofenac, cutaneous, gastrointestinal, cardiovascular, renal and laboratory parameters have been scrutinized for rates of adverse events. Placebo controlled trials of topical diclofenac in various non-OA musculoskeletal disorders found that a minority of patients experienced local skin reactions including dryness, rash, and pruritus, but not GI, cardiovascular or renal adverse reactions [53,55,57,58,59,60,61]. In the OA clinical trials, the majority have been in the knee OA patient group. Many of these trials have been placebo controlled (Table 3). 

Dreiser found a comparable rate of adverse events with skin as the only site of drug adverse reactions [38]. Grace also found comparable rates of mild adverse events in diclofenac treated patients compared to placebo group (6 *vs.* 9). Grace's placebo group was treated with the gel alone (without diclofenac) and most reactions were skin related, with one hirsutism and one nausea and abdominal cramping [40]. Brulmann reported one case of GI adverse reaction (nausea), and Baer found no difference between treatment and placebo groups in adverse reactions or withdrawals [39,47]. Niethard reported two diclofenac patients with GI adverse reactions compared to zero in the placebo group [41]. Roth compared topical diclofenac to vehicle, with no significant difference in GI adverse events or withdrawals between groups [44]. Bookman compared topical diclofenac to both vehicle and placebo, also reporting no significant differences between groups for GI events, or withdrawals [43]. In regards to cutaneous adverse effects, the topical diclofenac arm did have more dry skin reported in comparison to the vehicle control solution. The most common treatment adverse event reported in various studies was dry skin at the application site [62]. Application reactions including dry skin (27%), rash (12%) and pruritus (6%) were reported by Tugwell in his clinical trial of oral *vs.* topical diclofenac in knee OA patients. 

**Table 3:** Adverse effects reported in studies of topical diclofenac treatment for knee osteoarthritis.

Study	Treatment Arm	Control Arm	Adverse Effects—Comparing treatment and control
Dreiser 1993 [38]	Diclofenac hydroxyethylpyrrolidine (DHEP) plasters	Placebo plaster	Withdrawal due to adverse effect 0 in the treatment group and 1.3% in the placebo group (due to edema).
Grace 1999 [40]	2% Diclofenac in lecithin organogel	Placebo gel	GI: Nausea 2.9% *vs.* 5.9%.
			Cutaneous: Rash 10.5% *vs.* 14.7%, pruritus 0 *vs.* 2.9%, numbness 0 *vs.* 2.9%.
			Withdrawal due to adverse effect 2.9% in the treatment group (due to rash) and 0 in placebo group.
Bruhlmann 2003 [39]	DHEP patch	Placebo patch	GI: Nausea 1.9% *vs.* 0.
			Cutaneous: Rash 3.8% *vs.* 1.9%, pruritus 1.9% *vs.* 0.
			Withdrawal due to adverse effect 1.9% in the treatment group and 3.8% in the placebo group.
Bookman 2004 [43]	1.15% Diclofenac/45.5% DMSO	Placebo, Vehicle (45.5% DMSO)	Comparing topical diclofenac to placebo and vehicle.
			GI: Dyspepsia 7% *vs.* 6% *vs.* 5%, nausea 0 *vs.* 1% *vs.* 5%.
			Cutaneous: Dry skin 36% *vs.* 1% *vs.* 14%, rash 13% *vs.* 4% *vs.* 8%, paresthesia 14% *vs.* 6% *vs.* 22%, pruritus 11% *vs.* 4% *vs.* 8%.
			Other: Halitosis 5% *vs.* 0 *vs.* 1%.
			Withdrawal due to adverse effect 6.0% *vs.* 0 *vs.* 3.8%.
Roth 2004 [44]	1.15% Diclofenac/45.5% DMSO	Vehicle	GI: Abdominal pain 3.0% *vs.* 1.9%, dyspepsia 4.9% *vs.* 3.7%, melena 0 *vs.* 1.2%, nausea 2.4% *vs.* 0.6%.
			Cutaneous: Dry skin 36.6% *vs.* 25.3%, rash 11.0% *vs.* 4.9%, pruritus 0.6% *vs.* 0.
			Other: Halitosis 0 *vs.* 1.2%, taste perversion 1.8% *vs.* 3.1%.
			Withdrawal due to adverse effect 4.9% *vs.* 2.5%.
Tugwell 2004 [46]	1.5% Diclofenac/45.5% DMSO, placebo oral tablets,	Placebo topical solution, Diclofenac po 50 mg tid	GI: Abdominal pain 12% *vs.* 22%, dyspepsia 15% *vs.* 26%, melena 1% *vs.* 2%, nausea 8% *vs.* 13%.
			Cutaneous: Dry skin 27% *vs.* 1%, rash 12% *vs.* 2%, paresthesia 0.6% *vs.* 0.6%, pruritus 6% *vs.* 0.6%.
			Other: Halitosis 1% *vs.* 0.3%, taste perversion 2% *vs.* 0.6%, edema 7% *vs.* 8%, hypertension 1% *vs.*, 2%.
			Withdrawal due to adverse effect 20.6% *vs.* 25.4%
Baer 2005 [47]	1.5% Diclofenac solution/45.5% DMSO	Vehicle	GI: Abdominal pain 3.7% *vs.* 0.9%, dyspepsia 3.7% *vs.* 0.9%, gastritis 0.9% *vs.* 0, melena 0 *vs.* 0.9%, nausea 0.9% *vs.* 1.8%.
			Cutaneous: Dry skin 39.3% *vs.* 21.1%, rash 1.9% *vs.* 3.7%, paresthesia 1.9% *vs.* 1.8%, pruritus 0 *vs.* 2.8%. Other: Halitosis 1.9% *vs.* 0, taste perversion 3.7% *vs.* 1.8%.
			Withdrawal due to adverse effect 8.4% *vs.* 8.3%
Niethard 2005 [41]	1.16% Diclofenac diethylamine gel	Placebo gel	Overall GI adverse effect 0 *vs.* 1.7%.
			Overall cutaneous adverse effect 3.4% *vs.* 2.5%.
			Withdrawal due to adverse effect 1.7% *vs.* 1.7%
Barthel 2009 [42]	1% Diclofenac sodium gel	Vehicle	GI adverse event 5.9% *vs.* 5.0%.
			Cutaneous: Dermatitis 4.3% *vs.* 1.7%, skin dryness 0.4% *vs.* 0.8%, pruritus 1.6% *vs.* 0.4%.
			Withdrawal due to adverse effect 5.1% *vs.* 3.8%.
Simon 2009 [45]	1.15% Diclofenac/45.5% DMSO	Placebo solution, DMSO vehicle, Oral Diclofenac (100 mg SR) Topical + oral diclofenac	Comparing topical diclofenac, placebo, vehicle, oral diclofenac, and topical and oral diclofenac groups.
			GI: Abdominal pain 3.2% *vs.* 0.6% *vs.* 3.1% *vs.* 7.3% *vs.* 2.0%, dyspepsia 2.6% *vs.* 3.8% *vs.* 3.7% *vs.* 4.0% *vs.* 3.3%, nausea 0 *vs.* 0 *vs.* 0.6% *vs.* 2.0% *vs.* 3.3%.
			Cutaneous: Dry skin 18.2% *vs.* 3.2% *vs.* 11.2% *vs.* 2.6% *vs.* 19.7%, rash 2.6% *vs.* 0 *vs.* 3.1% *vs.* 0 *vs.* 0, pruritus 1.3% *vs.* 0 *vs.* 0 *vs.* 0 *vs.* 0.7%, contact dermatitis with vesicles 1.9% *vs.* 0 *vs.* 0 *vs.* 0.7% *vs.* 3.9%.
			Other: Abnormal taste 0 *vs.* 0.6% *vs.* 0.6% *vs.* 0 *vs.* 0.7%.
			Withdrawal due to adverse effect 10.4% *vs.* 11.5% *vs.* 7.5% *vs.* 12.6% *vs.* 15.1%

He found the topical preparation users had fewer GI adverse events (35% *vs.* 48%). This included abdominal pain in 12% of the topical group and 22% of the oral group, diarrhea in 9% *vs.* 17%, dyspepsia in 15% *vs.* 26%, melena in 1% *vs.* 2%, nausea in 8% *vs.* 13%. They also had fewer withdrawals due to GI events (6% *vs.* 16%). The topical treatment group was found to have fewer patients developing abnormalities in the hepatic transaminases, hemoglobin, and renal function [46].

Simon compared topical diclofenac with DMSO to topical placebo, topical DMSO vehicle, and oral diclofenac in a knee OA population and reported 6.5% GI adverse effects in the topical diclofenac treatment group which was not greater than placebo, but lower than the 23.8% in the oral diclofenac group. The majority of the GI events were abdominal pain, dyspepsia, and liver function test abnormality. There was one case of rectal hemorrhage in the topical diclofenac group. Hypertension was reported at a similar rate in all groups [45]. Simon also found that skin adverse effects were more frequently reported in the topical diclofenac group with again dry skin the most common event and the DMSO vehicle group having a skin adverse events rate in-between the topical diclofenac and placebo arms of the study. Shainhouse reported an open label study on topical diclofenac with DMSO in treatment of knee OA. They found that 45.1% of patients had adverse cutaneous reactions, including dry skin, contact dermatitis and contact dermatitis with vesicles. GI events were reported in 12%, and included gastroesophageal reflux, diarrhea, dyspepsia, nausea, abdominal pain, liver function test abnormalities, and GI bleeding in 8 patients (1%). Cardiovascular events were reported for 9.1% of patients and included angina, palpitations, myocardial infarction (0.5%), arrhythmia, venous thrombosis, and hypertension (3.5%). An elevation in creatinine was observed in 4.2% of patients participating [63].

In patients with hand OA, Zacher compared topical diclofenac to oral ibuprofen and found a comparable rate of adverse events overall (22% *vs.* 27%). The topical diclofenac group had a lower rate of GI adverse events (9% *vs.* 14%), and a lower rate of withdrawals (1.2% *vs.* 8.3%) [53]. In a more recent study Altman found 52% of patients receiving topical diclofenac sodium gel reported adverse events, compared to 43.9% in the vehicle comparison group. The incidence of GI adverse events was 7.6% in the treatment group and 3.7% in the vehicle group, with diarrhea the most common symptom reported and no GI bleeds. Headache was reported by 11% of the treatment group and 10% of the vehicle control group [52].

## 5. Conclusions

Both international and national specialty groups have published guidelines for the management of osteoarthritis. The American College of Rheumatology (ACR) and the European League Against Rheumatism (EULAR) guidelines suggest that topical NSAIDs be used if other oral pharmacologic strategies for the treatment of osteoarthritis have already been tried [16,17]. The Osteoarthritis Research International (OARSI) recommendations (which were published later than the ACR and EULAR reports) includes the guideline that topical NSAIDs can be effective as adjunctive or alternative therapy in the treatment of osteoarthritis of the knee [15]. The topical diclofenac studies show similarity in study design, duration and outcome assessments. For symptomatic osteoarthritis of the knees, there is evidence through several randomized studies that topical diclofenac is an efficacious treatment compared to placebo for a short duration with clinical efficacy appearing at within the first week and duration of effect up to 12 weeks. The clinical efficacy of topical diclofenac is likely secondary to local concentration when applied. Local cutaneous reactions are the most common adverse effects of topical diclofenac while the safety profile of systemic side effects is reduced compared to oral NSAIDs. Future research is required as there are no long term studies looking at the efficacy and safety for chronic (>12 weeks) use of topical diclofenac. It would be beneficial to have more evaluation and clinical trial comparison of the different topical diclofenac formulations to each other as well as to other topical NSAID preparations. It may be that certain formulations may be more advantageous in certain populations with their absorption and adipose tissue concentration that could allow for directed therapy.
